# Contribution of sewage to occurrence of phosphodiesterase-5 inhibitors in natural water

**DOI:** 10.1038/s41598-021-89028-3

**Published:** 2021-05-04

**Authors:** Youngmin Hong, Ingyu Lee, Beomseok Tae, Wonseok Lee, Shu-Yuan Pan, Seth W. Snyder, Hyunook Kim

**Affiliations:** 1 Technical Research Center, Shimadzu Scientific Korea, Gasan digital 1-ro, Geumcheon-gu, Seoul, 14508506 Korea; 2 Department of Environmental Engineering, University of Seoul, 163, Seoulsiripdae-ro, Dongdaemun-gu, Seoul, 02504 Korea; 3 Department of Chemical Engineering, Hankyong National University, 327, Chungang-ro, Anseong-si, Kyonggi-do, 17579 Korea; 4 Division of Waste-To-Energy Research, National Institute of Environmental Research, 42, Hwangyeong-ro, Seo-gu, Incheon, 22689 Korea; 5 Department of Bioenvironmental Systems Engineering, National Taiwan University, No 1, Sec 4, Roosevelt Rd., Taipei, 10617 Taiwan, R.O.C.; 6 McCormick School of Engineering, Technological Institute, Northwestern University, 2145 Sheridan Road, Evanston, IL 60208 USA; 7 Energy and Environment Science and Technology, Idaho National Laboratory, 1955 N Fremont Avenue, Idaho Falls, 83415 USA

**Keywords:** Environmental sciences, Environmental social sciences

## Abstract

Phosphodiesterase-5 inhibitors (PDE-5i, such as Sildenafil, Tadalafil and Vardenafil, mainly prescribed to treat erectile dysfunction) and their generic drug equivalents have been widely marketed and consumed in Korea. From the concentrations detected in wastewater, we could deduce that relatively large amounts of PDE-5i were consumed without a legal prescription. Thus, PDE-5i’s presence in the environment via sewage is unavoidable, and their environmental fate within a sewage treatment plant (STP) should be evaluated. In this study, we investigated the occurrence of three PDE-5i analogs in the influent and effluent of two STPs and the receiving water bodies. The PDE-5i concentration in total reached 62 ± 12 (STP#1) and 88 ± 37 ng L^−1^ (STP#2) in the sewage influent; about 70% of it was Sildenafil in both STPs. However, they were hardly removed by the STPs as the removal efficiency of the STPs was less than 10% ± 5%. Therefore, the pharmaceuticals were detected in the receiving water (lower than 7 ng L^−1^as a total amount) and the concentration slightly increased downstream of the STPs. A simple mass balance model applied for the compounds in the STP effluent and receiving water bodies also confirmed that the discharged PDE-5i were quite persistent. Lastly, we identified temporal and regional patterns in the consumption of the drugs from daily variations of PDE-5i in the influent to these two STPs. For instance, the levels of PDE-5i in the sewage significantly increased on weekends (from Friday to Saturday), and especially in the area where adult-entertainment businesses are common. We estimated that the amount of PDE-5i consumption in this area was 31% higher than that in the area with fewer nightlife spots. Considering that they are pharmaceutically active and resistant to treatment processes within an STP, it is advised that a regular monitoring and management program for PDE-5i should be developed to prevent the discharge of the pharmaceuticals into the water environment.

## Introduction

Phosphodiesterase-5 inhibitors (PDE-5i) have been widely used as a treatment for erectile dysfunction (ED)^1^, despite originally developed as the treatment for pulmonary hypertension, ischemic heart disease, and altitude sickness^2^. It was estimated that there would be approximately 322 million ED patients worldwide in 2025^3^. In particular, about 23% of male aged 30–39 years in Korea are currently suffering from ED^4^. Among PDE-5i analogues, Sildenafil (Viagra by Pfizer, USA), Tadalafil (Cialis by Lilly, USA), and Vardenafil (Levitra by Bayer, Germany) are the most widely prescribed^5,6^. The prevalence of ED in Asia is projected to increase from 87 million in 1995 to 200 million in 2025 due to the population aging^3^, and the global market for potential ED treatments is expected to reach $3.2 billion by 2022^7,8^. In the U.S., the largest market for PDE-5i, Viagra and its generic equivalents is estimated as much as $2.2 billion in 2018 (Viagra is about 10% of the total)^9,10^. In Korea, the market for Sildenafil increased from $33 million in 2012 (Viagra's patent expired) to $45 million in 2019. The total PDE-5i sales of the top 20 Korean pharmaceutical companies reached $133 million in 2019^11^.

Due to their effectiveness in treating ED and improving male sexual performance, PDE-5i are often illegally used as an ingredient of tonic medicines, diet supplements, and herbal dietary products in many countries (for example, U.S.^11^, EU^12^, Singapore^13^, Malaysia^14^, Korea^15,16^), thereby causing great public concerns^17–19^. A recent study found PDE-5i and their analogues in 80 out of 188 foods and dietary supplements marketed in Korea^15^. Moreover, since the expiration of patent protection for Sildenafil and Tadalafil in 2012 and 2015, respectively, a number of pharmaceutical companies worldwide have been producing generics and supplying them at a lower price^20^. Unapproved analogues are also illegally produced and sold without a proper prescription. Consequently, leaks of these pharmaceuticals into the water environment is expected^21^.

PDE-5i have been frequently detected in the influent of sewage treatment plants (STPs)^20,22–24^, as well as in the effluent at significant levels (as high as 349.6 and 28.6 ng L^−1^, respectively)^25^. As the cases of other pharmaceutically active agents, PDE-5i would exhibit adverse impacts on aquatic life after discharge into the environment, which was confirmed by others^26,27^. In fact, a few studies have reported the potential ecotoxicity of PDE-5i. For example, Temussi et al.^26^ reported that Sildenafil caused *Ceriodaphnia dubia* a chronic toxicity at sub-mg L^−1^ levels (EC_50_ = 0.64 mg L^−1^) and showed a distinct mutagenic and genotoxic potential at 2‒15 mg L^−1^. Similarly, other studies indicated that Sildenafil could cause phytotoxicity on germination and growth of terrestrial plants (*Lactuca sativa*, *Daucus carota* and *Lycopersicon esculentum*)^28^ and could even affect the fertility of aquatic organisms (*Asterias rubens* and *Psammechinus miliaris*) at environmentally relevant levels^29^. The predicted no-effect concentration of Sildenafil was estimated to be 640 ng L^−1^^27^. These reports raised concerns about the effect of these compounds on the aquatic ecosystem, especially due to unknown loads from their non-prescribed consumption.

Considering that PDE-5i are consumed for enhancing male sexual performance, their occurrence in sewage and eventually in the natural waters may largely depend on people’s lifestyle^30^. In other words, these compounds may be consumed more frequently at night than during the day, as well as in areas where adult-entertainment businesses are booming or they are distributed illegally. However, the pattern of PDE-5i spatial–temporal occurrence in urban areas has not been investigated. Therefore, we investigated the occurrence of PDE-5i in the urban sewer system during a day and over a week and their impacts on their receiving waterbody. The investigation consists of the following specific studies. First, we evaluated how the spatial–temporal consumption trends of PDE-5i according to lifestyle affect urban sewage. From the Seoul metropolitan area (the capital of Korea with a population of about ten million), two catchment areas were selected for the study; one is in the northern side of the Han River and another in the southern side. More commercial districts including adult-entertaining businesses (e.g., bars and clubs) have developed in the southern side (Supplementary Table S1). In addition, the gross regional domestic product per capita of the catchment area in the south is about 1.6 times of that of the northern catchment area. Each of the catchments is served by an STP; one (STP#1) for the catchment in the north and the other (STP#2) for the one in the south. The temporal and spatial distribution of PDE-5i in the city area was compared by investigating the concentration changes of the pharmaceuticals in the sewage flowing into the two STPs over time. Second, we investigated the fate of PDE-5i in the urban sewer system by monitoring the amounts of the pharmaceuticals flowing into and out of the STPs. This interpretation was performed including an estimation of the removal efficiency of each STP. Finally, we evaluated the persistence of PDE-5i in the water environment by investigating the distribution of the pharmaceuticals along the Han River that flows through the Seoul metropolitan area and receives discharges from the STPs via tributaries. For the purpose, a simple mass balance model was set up for the waterbody.

## Results and discussion

### Occurrence of PDE-5i in urban sewer system

**Figure 1:**
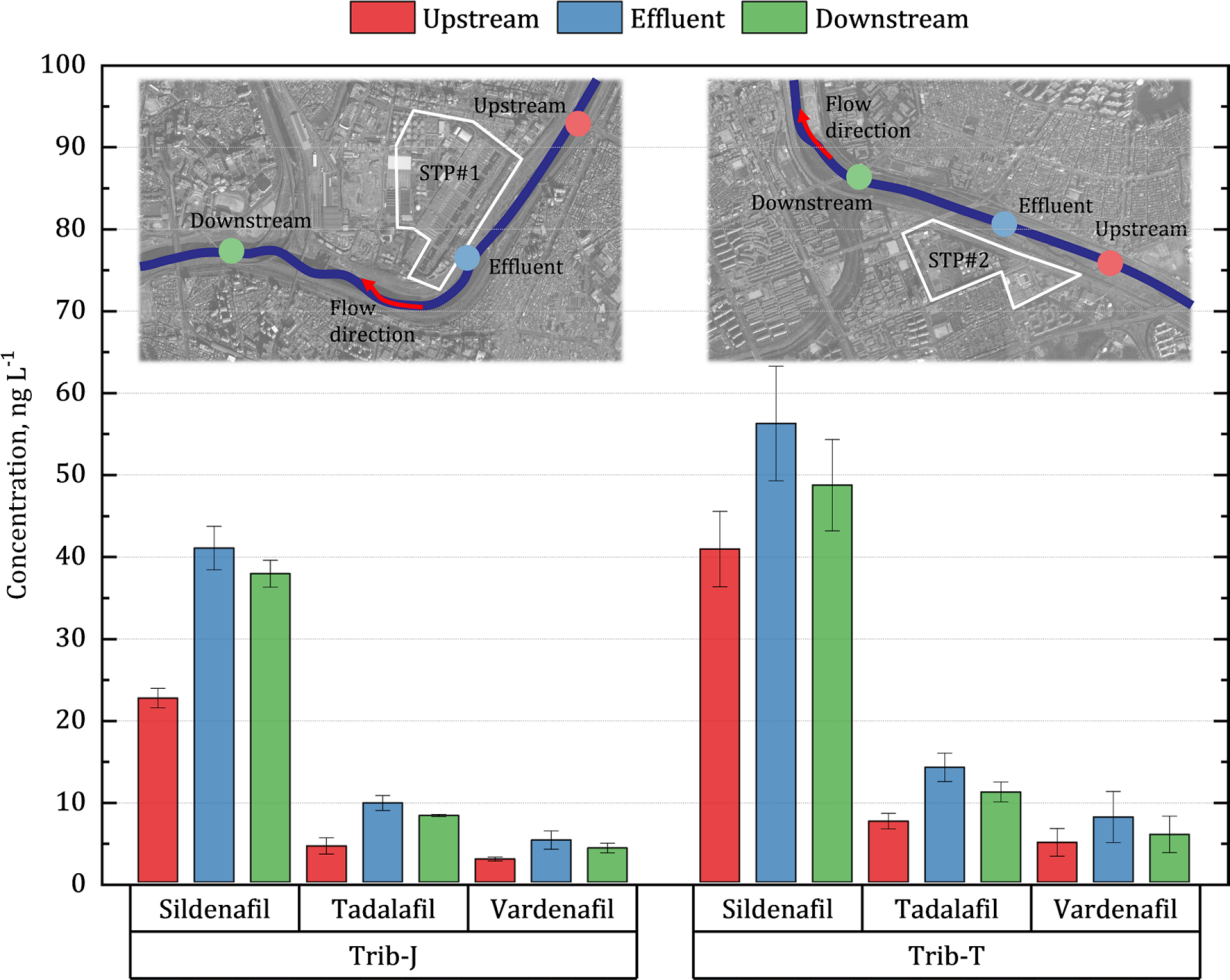
PDE-5i concentrations of effluent from STP#1 (from Trib-J) and STP#2 (from Trib-T) and water samples collected upstream and downstream of STPs. Satellite images obtained from the National Geographic Information Institute of Korea (http://map.ngii.go.kr).

As described in detail below, influent and effluent samples were collected from two STPs in Seoul, Korea (See supplementary Fig. S1); STP#1 is located at the north and the other (STP#2) in the south. The PDE-5i in sewage samples collected at the influent channel of two STPs are presented in Table 1. The sum of selected PDE-5i concentrations of the influent collected from STP#1 and STP#2 were 62 ± 12 and 88 ± 37 ng L^−1^, respectively, showing the regional difference (*n* = *7*, *p* < 0.05). Regionally, the PDE-5i concentrations of the water samples collected from the downstream of STP#2 (Trib-T) were higher than those of STP#1 (Trib-J) (Fig. 1). The concentration profiles of PDE-5i measured in the areas served by STP#1 and STP#2 were similar. The concentration profiles of PDE-5i along the tributaries confirm the contribution of STPs to PDE-5i contamination of the receiving waters is significant. This is since the effluent of STP#2 contained higher PDE-5i than that of STP#1.

**Table 1:** Concentration, mass balance, and removal efficiency of three PDE-5i measured in the Han River and its tributaries including the two STPs.

	Site	Flow rate	Sildenafil	Tadalafil	Vardenafil
		m^3s^ d^−1^	ng L^−1^	ng L^−1^	ng L^−1^
Han River	HR-1		2 ± 1	1 ± 1	1 ± 0.3
	HR-2		3 ± 2	2 ± 1	2 ± 0.2
	HR-3		3 ± 1	1 ± 1	2 ± 1
	HR-4		5 ± 2	2 ± 1	2 ± 1
	HR-5		4 ± 2	1 ± 0.3	1 ± 0.2
STP#1	Upstream	250,000	23 ± 3	4 ± 3	3 ± 1
	Influent	1,210,000	46 ± 7	10 ± 2	6 ± 3
	Effluent		41 ± 7	10 ± 2	5 ± 3
	Downstream	1,460,000	38 ± 4	8 ± 0.3	4 ± 2
	Downstream, estimated*		37 ± 5	9 ± 0.4	4 ± 3
	Mass balance, %**		102 ± 7	95 ± 7	102 ± 22
	Removal Efficiency, %		11 ± 4	2 ± 7	12 ± 3
STP#2	Upstream	510,000	41 ± 12	7 ± 3	5 ± 4
	Influent	750,000	62 ± 20	17 ± 7	9 ± 10
	Effluent		56 ± 19	14 ± 5	8 ± 8
	Downstream	1,260,000	49 ± 15	11 ± 3	6 ± 6
	Downstream, estimated*		50 ± 16	11 ± 4	7 ± 7
	Mass balance, %**		98 ± 3	99 ± 9	92 ± 20
	Removal Efficiency, %		10 ± 2	15 ± 3	11 ± 3
	Mean mass balance at STPs' downstream, %	100 ± 5	97 ± 8	97 ± 20	
	Mean removal efficiency of PDE-5i, %	11 ± 3	9 ± 9	11 ± 3	

*In order to confirm that the contribution of PDE-5i at the tributary was due to the discharge from the STP, the estimation was performed by reflect the flow rate at each sampling point.

**Measured using Eq. (2).

The mean concentrations of each ED drug in the influent were in the following order: Sildenafil (46 ng L^−1^ for STP#1 and 62 ng L^−1^ for STP#2) > Tadalafil (10 ng L^−1^ for STP#1 and 17 ng L^−1^ for STP#2) > Vardenafil (6 ng L^−1^ for STP#1 and 9 ng L^−1^ for STP#2). The order was also kept for the receiving water body as discussed below, although the levels were much less. It is worth noting that our measured levels of PDE-5i (especially, Sildenafil and Tadalafil) in the STP influents were somewhat higher than previous reports^22,24,31^. For instance, Baker and Kasprzyk-Hordern^22^ reported that the average concentration of Sildenafil in the influent samples collected from seven STPs in the UK was 24.9 ng L^−1^. Nieto et al.^31^ found that the average concentrations of Sildenafil, Tadalafil, and Vardenafil in 24-h composite samples collected from the influent of an STP in Spain were 31, 13, and 8 ng L^−1^, respectively (estimated from the published graphs).

The relatively higher concentrations of Sildenafil and Tadalafil observed in this study might be attributed to the expiry of patent-protection; patent-protection for Sildenafil and Tadalafil expired in 2012 and 2015, respectively, in Korea^32^. Since the patent-protection of Sildenafil and Tadalafil expired, about 60 pharmaceutical companies in Korea have produced 64 generic versions of Sildenafil and 160 versions of Tadalafil and marketed them at lower prices (as low as 20% of the original pharmaceuticals). Therefore, it is easy to conjecture that consumption of the pharmaceuticals are increasing^33^ and discharged into wastewater. In support of this conjecture, Causanilles et al.^20^ found that the concentration of Sildenafil in urban wastewater increased threefold from 2012 to 2015. Interestingly, the level of Vardenafil detected in sewage was significantly lower than those of Sildenafil and Tadalafil (see Table 1) both in this study and in the literature, which is expected with the consideration of its lower market share.

### Spatial–temporal variation of PDE-5i reflecting their local consumption patterns

**Figure 2:**
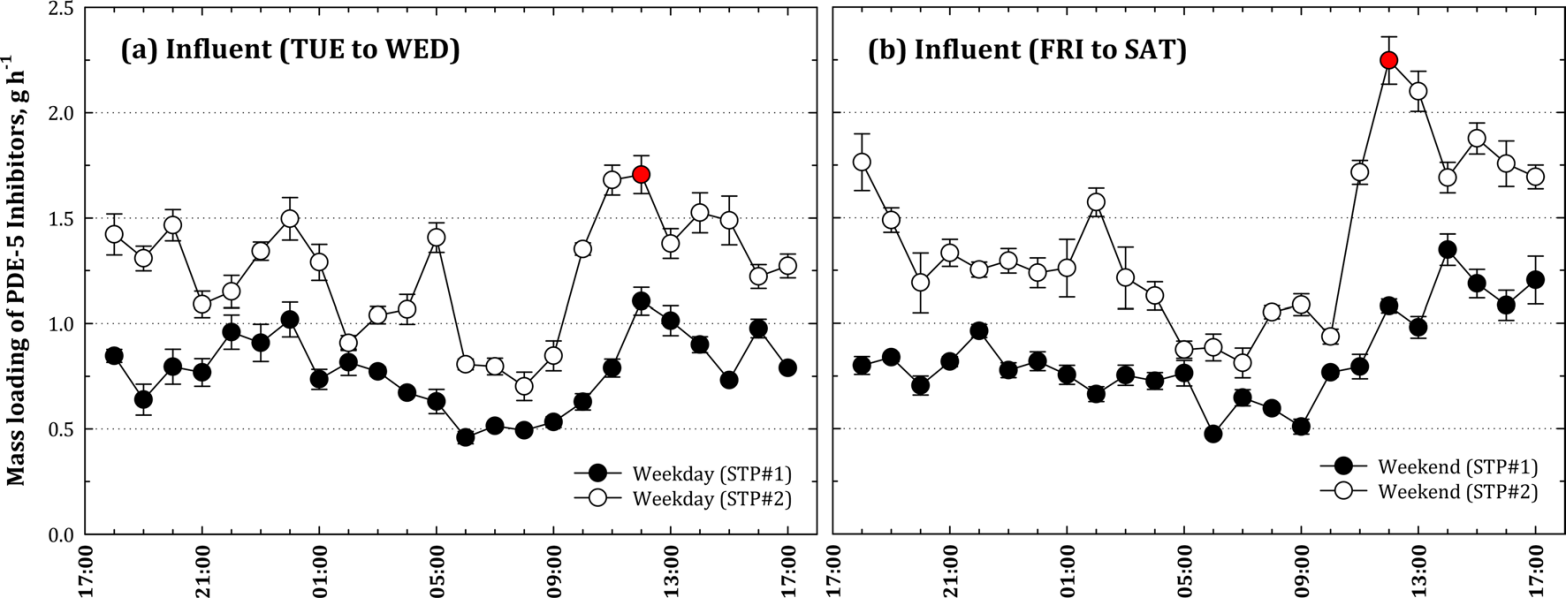
24-h profiles of PDE-5i mass loading on STP#1 and STP#2 on (**a**) weekday and (**b**) weekend. Red circles indicating peak of mass loading observed at STP#2 on weekday and weekend.

To investigate the spatial–temporal occurrence of PDE-5i, the influent water samples were collected hourly for 24 h from the two STPs; the levels of PDE-5i occurring at two different regions on a weekday and weekend were investigated. In general, if PDE-5i are consumed by users at night, they would be discharged into sewer in the following morning. The PDE-5i result in distinct differences of their elimination half-lives, 3–5 h for Sildenafil and 4–5 h for Vardenafil compared to 17.5 h for Tadalafil.^34^ We expected these pharmaceuticals that frequently consumed at night-time and excreted at the early morning (Fig. 2). In our investigated areas, the maximum time of arrival to reach STP#1 and STP#2 is about 6 and 3 h, respectively. Therefore, PDE-5i were expected to occur at the STPs about 12 h after their consumption. Temporally, the mean mass loading of PDE-5i on weekend was calculated higher than on weekday at both of STP#1 and STP#2. Regionally, the mass loading of PDE-5i on STP#2 was higher than that on STP#1. The difference between influent PDE-5i concentrations measured at STP#1 and that at STP#2 demonstrates the regional disparity of the drug uses. The mass loading of PDE-5i was calculated by multiplying hourly flowrate of each STP to the average of influent concentration.

**Figure 3:**
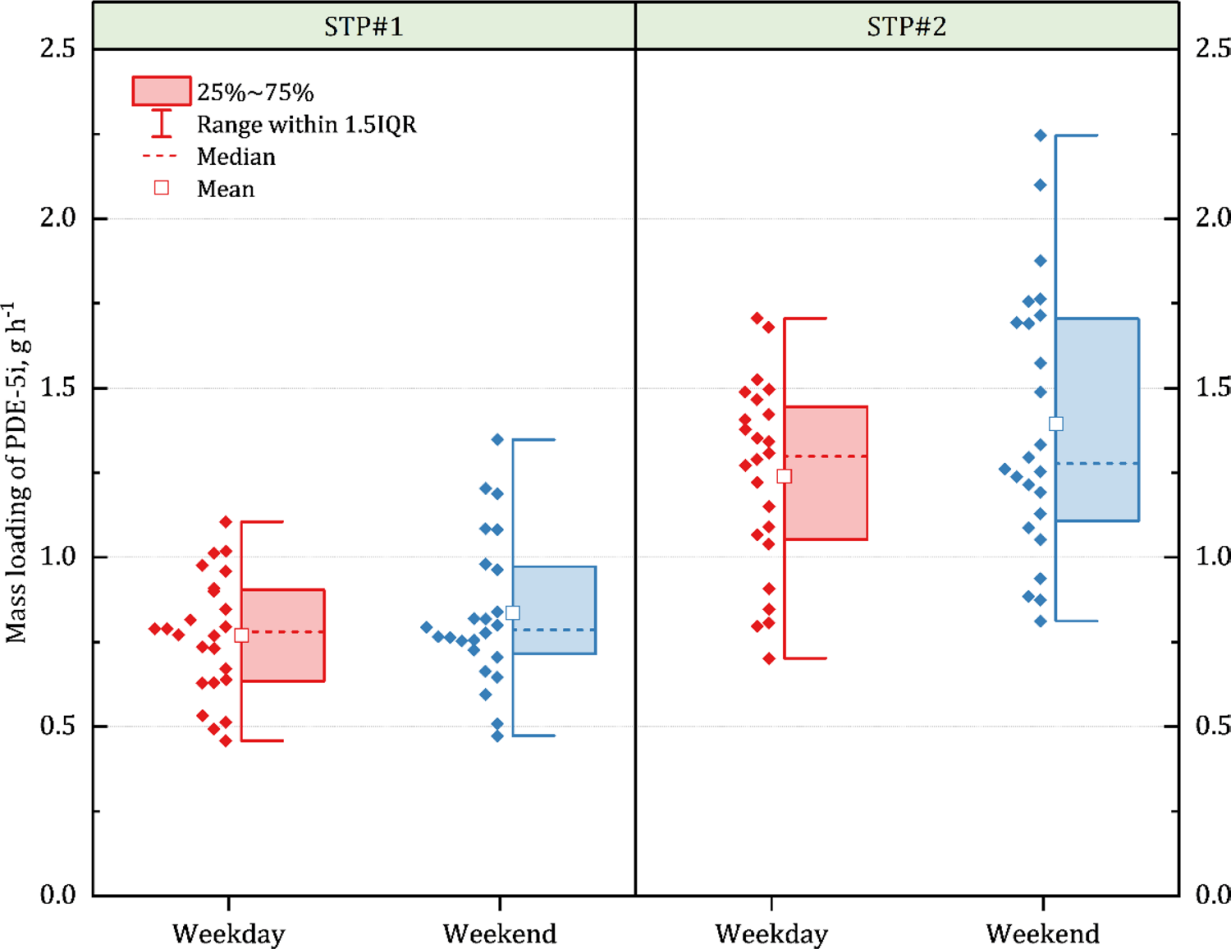
Boxplots for PDE-5i mass loadings on STP#1 and STP#2 calculated for weekday and weekend. Since no datum point was off the range for each STP set by 1.5 × IQR (inter-quartile range) and both mean and median are located within IQRs, the PDE-5i data are considered as normally distributed. For both weekday and weekend, PDE-5i mass loadings on STP#2 are higher than those on STP#1.

The temporal variations of PDE-5i concentration of the influents to STP#1 and STP#2 were evaluated as shown in Fig. 3. Supplementary Fig. S2 illustrates the total PDE-5i concentrations measured on the weekday and on the weekend. A statistically significant difference in PDE-5i mass could be identified between the weekday and the weekend at both STPs; the levels of PDE-5i measured on the weekend were higher than those measured on the weekday (*p* < 0.05). The concentrations of PDE-5i detected in the influent of both STP#1 and STP#2 on weekend (Friday-Saturday) were about 10‒22% higher than those detected on weekday (Tuesday-Wednesday). This observation implies that these pharmaceuticals might be consumed more on weekends than on weekdays. Regionally, the influent of STP#2 (from the districts of nightlife spots and clubs) contained a significantly higher concentration of PDE-5i (*p* < 0.01) than that of STP#1, implying that more PDE-5i might be consumed in the districts of nightlife spots and clubs. In other words, the use of the pharmaceuticals might be correlated with the district businesses of a city, as reported by Causanilles et al.^20^.

**Figure 4:**
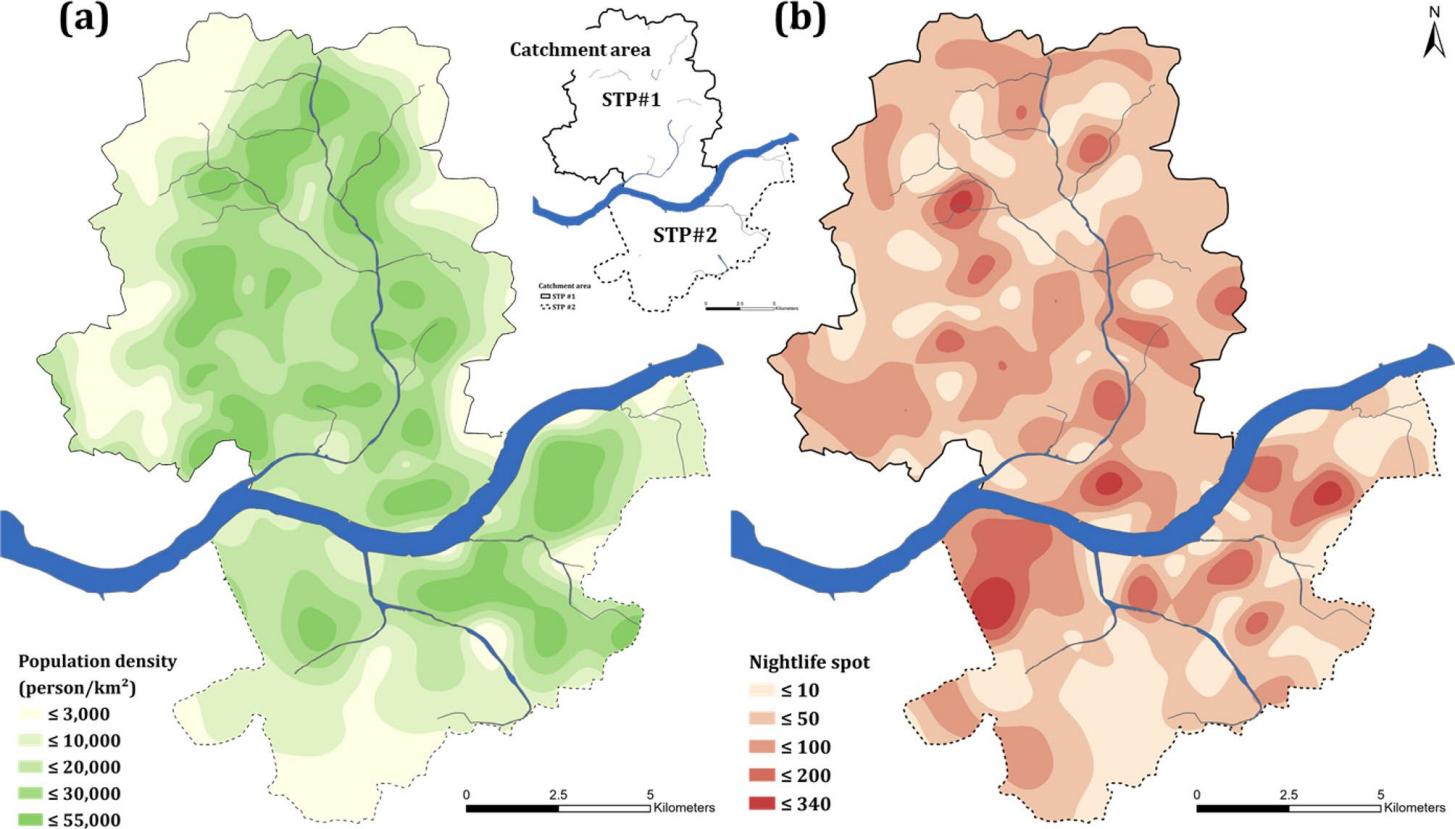
Contour map explaining (**a**) population density (colored green) and (**b**) nightlife spots (colored red) of areas served by STP#1 and STP#2. Geographic data obtained from the National Geographic Information Institute of Korea (http://map.ngii.go.kr) and processed using ArcGIS Pro (version 2.5; http://esri.com).

Based on the above analyses, we estimated that the total amount of PDE-5i consumed by 1,000 potential users on the weekday would be 205 and 268 mg for STP#1 and STP#2, respectively (Table 2). About 30% more PDE-5i were consumed in the STP#2 area, where more nightlife spots are located, than the STP#1 area (Fig. 4). The estimated amounts of Sildenafil consumed in both areas (i.e., 114 and 143 mg (1000 inhabitants)^−1^ for the STP#1 and STP#2, respectively) were about 1.3‒1.4 times and 12‒14 times higher than those of Tadalafil and Vardenafil, respectively. The higher consumption of Sildenafil in both areas clearly demonstrate the popularity of the chemical in the market. Recently, Causanilles et al.^35^ quantified the amounts of Sildenafil consumed in Brussels and Copenhagen; its consumption rates in Brussels and Copenhagen were calculated 74 and 78 mg (1000 inhabitants)^−1^, respectively. In fact, the Sildenafil concentrations measured in our study were approximately 3–4 and 2–5 times higher than those measured in Brussels (19 ng L^−1^) and Copenhagen (14 ng L^−1^), respectively.

**Table 2:** Amounts of PDE-5i consumed in areas served by STP#1 and STP#2 estimated by mass loading, hydraulic flow and bioavailability.

Catchment area	STP#1			STP#2		
Type of PDE-5i	Sildenafil	Tadalafil	Vardenafil	Sildenafil	Tadalafil	Vardenafil
Average concentration, ng L^−1^	53	12	6	62	16	6
Mass load, g d^−1^	65	15	7	45	11	5
Bioavailability, ratio*	0.40	0.81	0.15	0.40	0.81	0.15
Consumption by population, mg d^−1^ 1000 inh^−1^	114	82	9	143	114	10

*Francis and Corbin^43^

### Removal efficiencies of PDE-5i by the STPs

According to the aforementioned results, we observed that PDE-5i were regularly consumed and discharged into STPs. Here, we evaluated the performance of each STP in removing PDE-5i from wastewater by comparing the average PDE-5i concentrations of the influent and those of the effluent (Table 1). The results indicated that the removal efficiencies of PDE-5i by both STPs were not significant, and the two STPs did not show significant difference in the removal efficiency for each of PDE-5i (*p* < 0.05). For both STPs, the average removal efficiencies of Sildenafil, Tadalafil, and Vardenafil were only 11%, 9%, and 11%, respectively. The low removal efficiencies indicated that the current wastewater treatment technologies would be unable to handle the residual PDE-5i. The incapability of the two STPs in treating PDE-5i became more obvious when the removal efficiencies were calculated using the 24-h composite influent and effluent samples; the removal efficiencies were less than 10% (± 5%).

Based on the hypothesis that these compounds could be potentially removed from the wastewater by the adsorption onto the sludge in the STPs, we applied the BIOWIN model (EPI Suite ver. 4.11, U.S. EPA, Washington, DC, USA) for PDE-5i. However, the model predicted that only 2‒4% of Sildenafil, Tadalafil, or Vardenafil could be removed from wastewater by adsorption. The model also indicated that the removal of these compounds by the biodegradation mechanism would be minimal. We, therefore, evaluated the resistance of the pharmaceuticals to biodegradation by performing a few batch experiments, in which activated sludge collected from STP#1 had been applied for degrading Sildenafil and Tadalafil (Supplementary Fig. S3). In short, both Sildenafil and Tadalafil were degraded by less than 25% by activated sludge, even with a 15-h incubation. This result is also similar with removal efficiencies with Sildenafil concentration of 4 STPs in Korea.^36^ They were confirmed removal efficiencies of Sildenafil which was not degradation in the BNR process.

Only few studies have been reported in the literature in which the capability of an STP in degrading PDE-5i is evaluated (Table 3). The reported removal efficiency is quite inconsistent as shown in the Table 3: from below 15%^24,37,38^ to about 80%^25^. The large variation in reported removal efficiencies could be attributed to the additional treatment processes for the effluent from a biological process. Wastewater could be discharged without additional treatment or be disinfected using chlorine or other oxidants. It was reported that PDE-5i could be decomposed by chlorination^26^ or photo-oxidation^21,39^. Unlike the literature, PDE-5i were hardly degraded via chlorination in our study. It is because chlorine concentration of the effluent from the STPs was maintained at less than 0.15 mg Cl_2_ L^−1^ due to the public concern on the adverse impact of residual chlorine on the receiving water body. Nonetheless, none of these studies demonstrated that adsorption would contribute much to the removal of the pharmaceuticals; only less than 7% of the pharmaceuticals could be removed via adsorption^31^.

**Table 3:** Occurrences and removal efficiencies of PDE-5i by STPs reported by this study and the literatures.

Compounds	Influent, ng L^−1^	Effluent, ng L^−1^	Removal efficiency, % (Main process)	Sampling method	Time duration	References
Sildenafil	38–59, 49–86	32–52, 38–89	11 (MLE, A^2^O) 10 (MLE)	Grab	HRT*	This study
	32 (Mean)	8 (Mean)	74 (Activated sludge)	Composite	24-h	Nieto et al., 2010^31^
	15.0 (Median) 4.7–349.5	9.7 (Median) 5.1–28.6	40 (Trickling filter) 65 (Activated sludge)	Grab	–	Baker and Kasprzyk-Hordern, 2013^22^
	104 (Mean), n.d.**–517	62 (Mean), n.d.–289	0 (Activated sludge)	Composite	24-h	Papageorgiou et al., 2016^24^
	30	30	< 0 (Oxidation ditch and UV)	Grab	–	Sun et al., 2014^38^
	–	–	< 13 (Rotating biological contactor)	Grab	HRT	Delgado et al., 2018^37^
Tadalafil	8–13 12–24	8–12 8–20	< 5 (MLE, A^2^O) 15 (MLE)	Grab	HRT	This study
	n.d. ~ 20	n.d. ~ 1	81 (Activated sludge)	Composite	24-h	Nieto et al., 2010^31^
Vardenafil	2–11 3–21	2–11 2–20	10 (MLE, A^2^O) 11 (MLE)	Grab	HRT	This study
	n.d.–20	n.d.–1	80 (Activated sludge)	Composite	24-h	Nieto et al., 2010^31^

*HRT: Sampling interval between influent and effluent considered by hydraulic retention time.

**n.d.: not detected.

We also noticed that sampling of influent and effluent water for PDE-5i analysis in the literature did not consider the HRT of an STP, nor the behavior of consumers. For instance, the pharmaceuticals would be mainly consumed at night and then released into the sewer the next morning. Therefore, sampling campaigns should be carefully designed in consideration of consumer behavior and the HRT of STPs to avoid false interpretation^40^.

### Occurrence and persistence of PDE-5i in natural water environment

**Figure 5:**
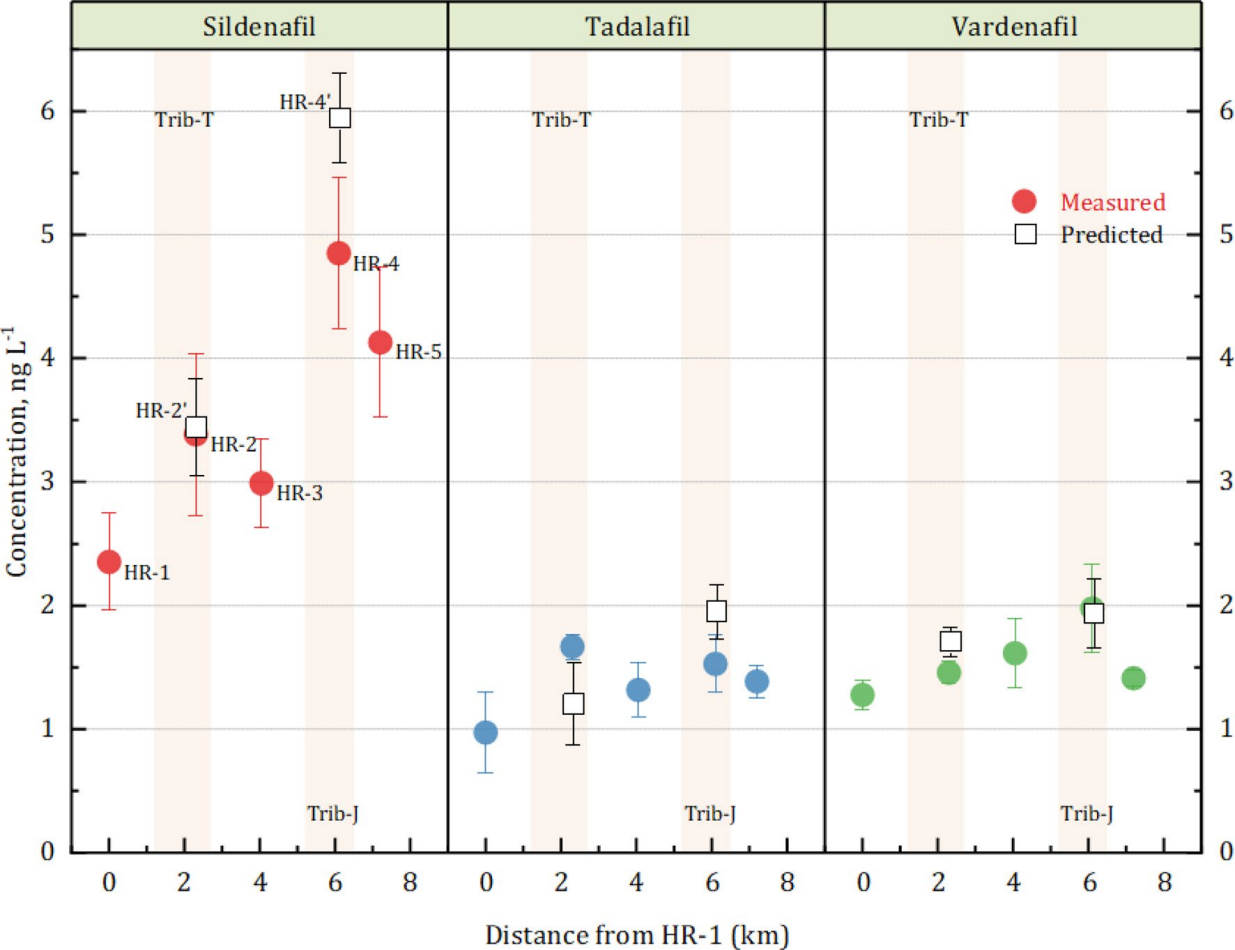
PDE-5i concentrations of water samples collected along Han River; HR-1 (utmost upstream) to HR-5 (utmost downstream).

Since we have found that PDE-5i would be hardly removed within an STP before they are discharged, it could be easily conjectured that they would be persistent in the environment. Therefore, we investigated the occurrences of PDE-5i in the mainstream (Han River) and in the tributaries (viz. Trib-J and Trib-T) that receive the discharge, as shown in Fig. 5. The two tributaries (Tri-T and Tri-J) merged near the HR-2 and 4 sampling points. The model estimation was made for PDE-5i concentrations at HR-2 and 4. The predicted values of HR-2 and 4 (denoted by rectangles) were similar with measured values; less than 15% errors were observed. This result demonstrated that the presence of PDE-5i in the mainstream was originated from the tributaries carrying the discharges of the STPs. Standard errors were evaluated using seven replicates.

Since we did not have any precipitation for a week before or during the sampling event and almost all wastewater generated in the catchment areas under study is collected by combined sewer systems and treated by STP#1 and STP#2 (Supplementary Table S1), nonpoint sources of the PDE-5i from urban runoffs were not considered. The results indicated that the sums of the average concentrations of three PDE-5i detected along the Han River (HR-1 to HR-5) were less than 7 ng L^−1^. The profiles of PDE-5i concentrations along the Han River exhibited a slightly increasing trend. This should be attributed to the mass-loading of PDE-5i from the two tributaries that receive the discharges of STP#1 and STP#2 and then join the Han River at HR-2 and HR-4, respectively (Fig. 5).

Since PDE-5i are hardly degraded in an STP before they are discharged and resistant to adsorption on particulate matters in water with their low Log K_OW_ (i.e., < 4.5), it is easily conjectured that they would be persistent in the environment. Nonetheless, the PDE-5i degradation mechanisms in the environment are still unknown; in fact, information pertaining to the concentrations of PDE-5i in natural waters is quite limited. Baker and Kasprzyk-Hordern^17^ observed Sildenafil in the effluent of an STP but could not detect the pharmaceutical downstream from the STP. However, this could be due to a high detection limit of their analytical method (4.6 ng L^−1^) in their study. In short, the presence of PDE-5i in the main stream originated from the tributaries carrying the discharges of the STPs. In conclusion, based on the observations, the PDE-5i detected in the natural water environment purely originated from wastewater produced by human activities.

To better illustrate the contribution of STPs to PDE-5i contamination of the receiving water body, we formulated a mass balance model (Eq. 1). It should be noted that the degradation of PDE-5i in the natural water was not considered in the model because the engineered treatment systems was not effective at eliminating the drug. The mass loadings of PDE-5i in the downstream of tributaries after STP#1 and STP#2 during the sampling were calculated: they were 10 and 3 times higher than those detected in the upstream of STP#1 and STP#2, respectively. Table 1 summarizes the % ratios of the estimated and measured concentrations of PDE-5i in the tributaries downstream of each STPs. Based on the daily flow rates of the STPs and the measured flow rates of the streams, the ratios were calculated as 98% ± 12%. The results indicated that PDE-5i discharged from the STP should be barely degraded in the natural water environment. The concentrations of PDE-5i from the HR-1 to HR-5 (the mainstream) were also estimated in the same manner as described above and were compared with the measured concentrations. The ratio of PDE-5i calculated for HR-4 was found to be 106%, indicating that the PDE-5i discharged from STP#1 and STP#2 would directly flow into the main stream with little-to-no natural degradation. The concentrations of PDE-5i along the main stream gradually increased from 4 ng L^−1^ (at HR-1) to 7 ng L^−1^ (at HR-5) due to the wastewater discharged from these STPs. With the result, it was concluded that PDE-5i in the water environment might not be controlled unless PDE-5i in wastewater would be properly treated and prevented from discharge.

## Summary

To investigate the spatial–temporal distribution of PDE-5i (i.e., Sildenafil, Tadalafil, and Vardenafil) in the water environment, water samples were collected from two STPs and their receiving water bodies. Domestic wastewater was observed to contain ng L^−1^ levels of the PDE-5i. However, no significant reduction of PDE-5i by the STPs was observed. From the temporal point of view, interestingly, higher PDE-5i concentrations were observed in domestic wastewater collected on a weekend (Friday to Saturday) than on a weekday (Tuesday to Wednesday), implying that more PDE-5i were consumed on weekends. Spatially, the concentration of PDE-5i in the influent of STP#2 (serving for the drainage areas with nightlife spots and clubs) was 17% higher than that of STP#1 (serving for the residential areas). However, the estimation of the consumption rate of PDE-5i per 1000 adult male-residents showed a significant difference between the STP#1 area and the STP#2 area. The estimated consumption rates of PDE-5i were 205 and 268 mg d^−1^ (1000 inhabitants)^−1^ for STP#1 and STP#2, respectively; 31% more of the ED pharmaceuticals were consumed in the STP#2 area than in the STP#1 area. The results clearly indicate the potential relationship between the use of PDE-5i and the area of adult-entertainment businesses, which needs further research.

Nonetheless, the PDE-5i in domestic wastewater were barely treated by the STPs and, eventually, discharged into the water environment. In fact, they were not degraded and persistent in the receiving water, which was confirmed by a simple mass balance model. About 2% and 15% differences between the calculated and measured concentrations of PDE-5i were found for the tributaries downstream of the STPs and for the Han River, respectively. Considering the increasing market size, it is easily expected that more PDE-5i will be discharged in an urban area. The result from our study demonstrated that PDE-5i, which continuously flows into the water environment, would not be removed by the STP and be persistently conserved in the receiving water. Therefore, an integrated risk management plan associated with PDE-5i discharge in the water environment should be urgently designed and implemented.

## Methods

### Chemicals and reagents

Sildenafil citrate (CAS No. 171599-83-0), Tadalafil (CAS No. 171596-29-5), and Vardenafil hydrochloride (CAS No. 330808-88-3) standards were prepared with certified reference materials, which were purchased from the Sigma-Aldrich (St. Louis, MO, USA) and the United States Pharmacopeia (Rockville, MD, USA). Sildenafil-d_3_, widely used as a surrogate standard, was obtained from the Toronto Research Chemicals (North York, ON, Canada). Methanol, acetonitrile, water, and acetic acid (MS grade) were obtained from the Honeywell (Muskegon, MI, USA) and Sigma-Aldrich. Disodium ethylene diamine tetra acetic acid (Na_2_EDTA, ACS reagent grade) was also purchased from the Sigma-Aldrich.

Individual stock solutions (1 mg mL^−1^) were prepared by dissolving in methanol and then stored at less than 4 °C. Supplementary Table S2 presents the chemical structures and properties of each target compound. The mixtures of working solutions for the calibration were prepared by diluting the stock solution to desirable concentrations in a mixture of 0.1% acetic acid, 10% methanol, and 400 mg L^−1^ Na_2_EDTA.

### Preparation of water sample analysis

The collected water samples were immediately shipped to the laboratory, pre-treated, and stored in a refrigerator until being analyzed. For each sample, the total organic carbon (TOC), total nitrogen (TN), total phosphorus (TP), ammonium, phosphate, nitrate, and nitrite concentrations were analyzed immediately after the sample was shipped to the laboratory (Supplementary Fig. S4). Before the LC–MS/MS analysis, the water samples were filtered through a 0.2 μm polyvinylidene fluoride syringe filter (Advantec, Tokyo, Japan). Then, methanol (10%, v/v), acetic acid (0.1%, v/v), and 400 mg L^−1^ Na_2_EDTA were added to the filtrate. Once a sample is prepared for its analysis, 300 μL of it was injected into the LC–MS/MS system. All samples were analyzed in triplicate.

### Analytical method

Chromatographic analysis was performed using a liquid chromatography–tandem mass spectrometry (LC–MS/MS) system (LCMS-8050, Shimadzu, Kyoto, Japan) coupled to a solid-phase extraction (SPE) column (Shim-pack MAYI-ODS; 10 mm × 2.0 mm in diameter, 50 μm particles; Shimadzu, Kyoto, Japan)-integrated switching device. Supplementary Fig. S5shows the schematic diagram of the LC–MS/MS system. Water samples were injected into an Evolute Express ABN pretreatment column (30 mm × 2.1 mm in diameter, 20 μm particles; Biotage, Uppsala, Sweden) and eluted at a flow rate of 0.4 mL min^−1^ using 0.1% acetic acid in water. The time to trap the target compounds was set at 2.5 min. After the target compounds were trapped, the pretreatment column was back-flushed with a mixture of 0.1% aqueous acetic acid/acetonitrile/methanol/isopropanol (= 1/1/1/1, v/v/v/v) by switching the flow selection valve and then stabilized before the next sample was analyzed.

The analytes were separated using an ACE 5 PFP-C_18_ column (150 mm in length × 2.1 mm in diameter, packed with 5.0 μm particles; Advanced Chromatography Technologies, Aberdeen, UK) at a flow rate of 0.2 mL min^−1^. The temperature of the column oven was maintained at 40 °C. 0.1% acetic acid in water (A) and acetonitrile (B) were used as mobile phases for the LC. The following solvent gradient program was applied for separation of target PDE-5i: 10% B at 0–2.5 min; 10% to 100% B at 2.5–12 min; 100% B at 12–17 min, and 100% to 10% B at 17–20 min. All target analytes were quantified in multiple-reaction monitoring (MRM) mode using electrospray ionization in the positive ion mode. The flow rates of the nebulizing and drying gases (both were N_2_) and capillary heating gas (dry air) were 3, 10, and 10 L min^−1^, respectively. The capillary, desolvation, and heating-block temperature were maintained at 300, 250, and 400 °C, respectively. Detailed explanation of the analytical method can be found from the paper published by Hong et al.^30^ The MRM parameters were optimized with respect to collision energies (Supplementary Table S3).

To examine the validity of the analytical method used in the study, the linearity, method detection limit (MDL), recovery (%), and repeatability (%RSD, *n* = 7) were evaluated. The ranges of the calibration curve for each target compound were determined based on the sensitivity of the analytical instrument. The standard curves within the designated calibration ranges showed an excellent linearity (Supplementary Fig. S6; R^2^ > 0.99). The MDL for each target compound analyte was determined by multiplying the Student *t*-value by the standard deviation calculated using seven replicates of 5 ng L^−1^ of the analyte. The MDLs in this study were determined 0.3, 0.2, and 0.7 ng L^−1^ for Sildenafil, Tadalafil, and Vardenafil, respectively. Both recovery and repeatability were calculated based on the analytical results for samples spiked with the standard target analytes at three levels. For each target analyte at the desired level, the recoveries ranged from 81 to 100% for influent and effluent from the STP, while repeatability was below 10%. In addition, Sildenafil-d_3_ was selected as a surrogate standard and spiked at a level of 0.04 ng mL^−1^. The surrogate recovery of PDE-5i was represented 91.6 91.6% ± 5.2% (Supplementary Table S4). Moreover, the sample was reanalyzed whenever the accuracy of the surrogate standard added to a sample showed a difference of more than 25%. We provide the results of PDE-5i of water samples collected from STPs (including influents) and from stream waters based on our validated analytical methods.

### Water sampling points along the environmental water and at STPs

Water samples were collected at nine sampling sites: four sites along the two tributaries (denoted by red and purple circle dots) and five along the mainstream, which were denoted by yellow circle dots (Supplementary Fig. S1). Two tributary water samples were collected from the upstream and downstream of discharge points from the STPs denoted by star dots: one STP in the northern side of the Han River (STP#1) and the other in the southern side (STP#2). There are two tributaries merging into the Han River in our study area; one of which is Trib-J (in the northern side) and the other is Trib-T (in the southern side). Water samples were also collected upstream (denoted by red circle dots) and downstream (denoted by purple circle dots) of each tributary (Trib-J and Trib-T).

The water sampling campaign in this study was carried out as follows: First, the water samples of the tributaries containing STP were collected for 7 consecutive days in Spring (from 21 to 27 April). In this campaign, samples were collected from all sampling points (13 sites in total; 5 sites along Han River (HR-1∼5), 4 sites along the two tributaries, and 4 sites of influent and effluent from STP#1 and STP#2) except the Han River and tributaries using a composite sampler every 24 h (in each sampling event, three samples collected for triplicate analysis). The sampling campaign for the influent and effluent of the STPs was designed with the consideration of the time-lags (flowing time from the inlet and outlet of each STP). Effluent samples were collected from the outlet. Meanwhile, in the case of the Han River, we collected 1 L sample at each point while moving downstream using a boat. Second, to observe the diurnal variation of PDE-5i, we collected the influent and effluent water samples from the STPs on weekdays (Tuesday-Wednesday) and weekends (Friday-Saturday) in Jun. These samples were collected hourly for 24 h using a composite sampler, and 24 samples by date were analyzed in triplicate.

The STP#1 mainly consists of two biological nutrient removal (BNR) systems: the modified Ludzack-Ettinger (MLE; anoxic/oxic) and the A^2^O (anaerobic/anoxic/oxic) processes. Before discharged into the tributary, the effluents from both BNR systems are operation of disinfection of chlorination. However, in these STPs, there are operated intermittently chlorination treatment, but the contact time was less than 15 min. It may not affect the degradation of PDE-5is in this study.

The treatment capacity of STP#1 is about 945,000 m^3^ d^−1^, mainly sewage from residential areas, where the MLE and A^2^O processes have a capacity of 857,000 and 353,000 m^3^ d^−1^, respectively. In the case of STP#2, a BNR system based on the MLE process has a capacity of 750,000 m^3^ d^−1^. The plant treats wastewater from both residential and commercial areas. It is noticeable that adult-entertainment businesses are well-developed in the commercial areas served by STP#2 (see Supplementary Table S1)^41^.

### Experimental setup for biodegradation batch assays

Batch experiments for evaluating biodegradability of PDE-5i were carried out in a 1 L Pyrex glass reactor. Mixed liquor suspended solids (MLSS; concentration: 1500 mg L^−1^) collected from a lab-scale A^2^O system was used as inoculum. The batch tests were individually carried out only with Sildenafil and Tadalafil; each pharmaceutical of 1–1.6 μg L^−1^ was spiked into the reactor. Oxygen was supplied via a ceramic diffuser to maintain dissolved oxygen concentration at about 2 mg O_2_ L^−1^. The reactor was incubated at 20 °C and continuously stirred with a magnetic bar.

During the tests, aliquots were taken from the batch reactor and filtered using a syringe filter (0.2 μm PVDF; Advantec Co., Kyoto, Japan). Then, the filtered samples were analyzed to quantify the target compounds by LC–MS/MS. Dissolved organic carbon was measured by an on-line total organic carbon (TOC) analyzer (TOC-L, Shimadzu, Kyoto, Japan).

The Estimation Programs Interface (EPI) Suite and BIOWIN were applied for estimating the physical/chemical properties and aerobic and anaerobic biodegradability of PDE-5i. The estimation was compared with the result from the batch assay tests.

### Mass balance model for estimating PDE-5i concentration along river receiving discharge from STPs

We formulated a simple mass balance model based on the mass loadings of PDE-5i in the tributaries. We estimated the concentrations of PDE-5i detected in the tributary downstream of each STP^42^ using Eq. (1). The estimated PDE-5i concentrations were compared with the experimentally measured ones, which are presented as % ratio (Eq. 2):

1 $$C_{{DW\left( {estimated} \right)}} = \frac{{C_{UP} Q_{UP} \times \mathop \sum \nolimits_{i = 1,2} C_{{i_{EFF} }} Q_{{i_{EFF} }} }}{{Q_{DW} }}$$

2 $$Mass\,\, Balance \left( \% \right) = \frac{{C_{{DW\left( {measured} \right)}} }}{{C_{{DW\left( {estimated} \right)}} }} \times 100$$

where *C* is the concentration of PDE-5i (ng L^−1^), *Q* is the flow rate (m^3^ d^−1^), and the subscripts indicate sampling sites (UP: upstream, DW: downstream, and EFF: effluent from STP#1 or STP#2). Flow rates for STP discharges and the receiving river including tributaries are measured using on-line flow sensors.

### Estimation of PDE-5i consumption

Based on the PDE-5i concentrations in the influent for each STP, the consumption rates of PDE-5i in the two regions served by the STPs were estimated (Eq. 3). It was hypothesized that the pharmaceuticals would be consumed by adult male residents aged 30–69 and that un-metabolized portion of the pharmaceuticals would be discharged into sewer systems. The extent of the metabolism of each PDE-5i compound in a human body was obtained from the bioavailability data reported by Francis and Corbin^43^.

3 $${\text{Consumption}}\;\left( {{\text{mg}}\;{\text{d}}^{ - 1} \;1000\;{\text{inh}}^{ - 1} } \right) = \frac{{\mathop \sum \nolimits_{t = 1}^{24} \left[ {Concentration _{t} \left( {{\text{mg}}\;{\text{L}}^{ - 1} } \right) \times {\text{ Flow}}_{t} \left( {L {\text{h}}^{ - 1} } \right)} \right]}}{{(1 - {\text{Bioavailability}}) \times {\text{Population}} \left( {1000 \;{\text{inh}}} \right) }}$$

where *Concentration*_*t*_ is the PDE-5i concentration of the influent wastewater of each STP (ng L^−1^) at time *t*, *Flow*_*t*_ is the average hourly volumetric flow rate (m^3^ h^−1^) between each sampling interval (i.e., between *t* − 1 and *t*), and population is the number of potential PDE-5i consumers (inh) in the area serviced by each STP. Lastly, *Bioavailability* is the extent of metabolism of each PDE-5i in a human body.

### Statistical analysis

The statistical analysis and graphical presentation of the results in this study were made using Microsoft Office Excel 2016 (Microsoft Inc., Redmond, WA, USA) and Origin 2019 (OriginLab Corp., Northampton, MA, USA). For spatial–temporal comparisons, the differences between the levels of PDE-5i concentrations in water samples collected from STPs were evaluated using the paired *t*-test. The geographic data about the catchments under study were obtained from the National Geographic Information Institute (http://map.nagii.go.kr) and processed using ArcGIS Pro (version 2.5; Esri, Redlands, CA, USA; http://esri.com).

## Supplementary information

Supplementary information
