# Supplementary material for: Contribution of sewage to occurrence of phosphodiesterase-5 inhibitors in natural water
Source: Sci Rep. 2021 May 4;11:9470. doi: 10.1038/s41598-021-89028-3 (PMC8096833; doi:10.1038/s41598-021-89028-3)
Supplement: Supplementary file 1 — Supplementary information [file 41598_2021_89028_MOESM1_ESM.docx]

**Contribution of Sewage to Occurrence of**

**Phosphodiesterase-5 Inhibitors in Natural Water**

Youngmin Hong^1,†^, Ingyu Lee^2,†^, Beomseok Tae^3^, Wonseok Lee^4^, Shu-Yuan Pan^5^,

Seth W. Snyder^6,7^ and Hyunook Kim^2,*^

^1^ Technical Research Center, Shimadzu Scientific Korea, 145, Gasan digital 1-ro, Geumcheon-gu, Seoul 08506, Korea

^2^ Department of Environmental Engineering, University of Seoul, 163, Seoulsiripdae-ro, Dongdaemun-gu, Seoul, 02504, Korea

^3^ Department of Chemical Engineering, Hankyong National University, 327, Chungang-ro, Anseong-si, Kyonggi-do, 17579, Korea

^4^ Division of Waste-to-Energy Research, National Institute of Environmental Research, 42, Hwangyeong-ro, Seo-gu, Incheon 22689, Korea

^5^ Department of Bioenvironmental Systems Engineering, National Taiwan University, No 1, Sec 4, Roosevelt Rd., Taipei, 10617 Taiwan (ROC)

^6^ McCormick School of Engineering, Northwestern University, Technological Institute, 2145 Sheridan Road, Evanston, IL 60208, USA

^7^ Energy & Environment Science and Technology, Idaho National Laboratory, 1955 N Fremont Avenue, Idaho Falls, 83415, USA

^†^These authors contributed equally: Youngmin Hong and Ingyu Lee

^*^This author supervised this work: Hyunook Kim. E-mail: h_kim@uos.ac.kr

**Table S1 The operation parameters and sewerage information of STP#1 and STP#2.**

|  | STP#1 | STP#2 |
| --- | --- | --- |
| Capacity, m^3^ d^-1^ | 1,590,000 | 900,000 |
| Flowrate, m^3^ d^-1^ | 1,210,000 | 750,000 |
| Catchment area, km^2^ | 255.5 | 97.8 |
| Sewerage coverage, % | 100 | 100 |
| Service population (male, 30-69 years old), py | 3,385,000 (958,000) | 1,681,000 (528,000) |
| Main biological process | A^2^O^1)^, MLE^2)^ | MLE |
| Hydraulic retention time (Solids retention time), h | 8~10 (10~15) | 6~9 (6.5~10.5) |
| Registered adult-entertainment businesses^3)^ | 240 | 479 |
| Proportion of residential to commercial area^4)^, % | 92: 8 | 88.9: 8.3 |
| Gross regional domestic product per capita^4)^, USD | 35,116 | 57,393 |

^1)^ Anaerobic, anoxic and oxide; ^2)^ Modified Ludzack-Ettinger; ^3)^Data from Korean Statistical Information Service (accessed on Jan 10, 2021). ^4)^Data from Seoul Data Portal Service (https://data.seoul.go.kr; accessed on Jan 10, 2021).

**Table S2 Chemical structure and property of target compounds.**

| Property | Sildenafil | Tadalafil | Vardenafil |
| --- | --- | --- | --- |
| Structure | 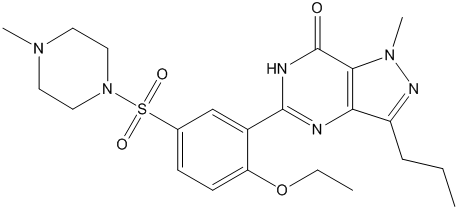 | 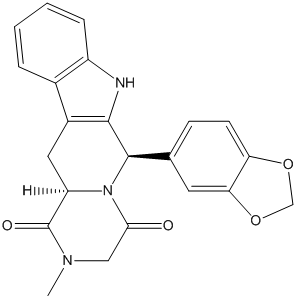 | 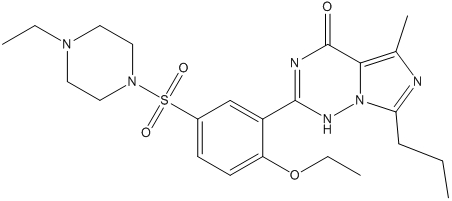 |
| Molecular formula | C_22_H_30_N_6_O_4_S | C_22_H_19_N_3_O_4_ | C_23_H_32_N_6_O_4_S |
| Molecular weight (g mol^-1^) | 474.6 | 389.4 | 488.6 |
| p*K*_a_ | 6.78 / 9.12^1)^ | -4.2 / 15.17^2)^ (Est.) | 6.21 / 8.01^2)^ (Est.) |
| Log *K*_ow_^3)^ | 2.75 | 1.42 (Est.) | 2.79 (Est.) |
| Solubility^3)^ (mg L^-1^ in Water) | 4.7 (Est.) | 220 (Est.) | 3.5 (Est.) |

^1)^Gorby et al. (2000); ^2), 3)^ChemAxon

**Table S3 MRM parameter.**

| # | Compound | Classification | R.T. | Quantitation ion | |  | Qualification ion | |  |
| --- | --- | --- | --- | --- | --- | --- | --- | --- | --- |
|  |  |  |  | Q1 Mass | Q3 Mass | CE | Q1 Mass | Q3 Mass | CE |
|  |  |  | min | m/z | m/z | V | m/z | m/z | V |
| 1 | Sildenafil | Target | 8.6 | 475 | 58 | -50 | 475 | 100 | -30 |
| 2 | Tadalafil | Target | 9.6 | 390 | 268 | -16 | 390 | 204 | -55 |
| 3 | Vardenafil | Target | 8.0 | 489 | 151 | -49 | 489 | 312 | -41 |

**Table S4 Method validation data of PDE5-i in this study**

| Compound | Spiked level, μg L^-1^ | | | Recovery, n=7, % | | | | | |
| --- | --- | --- | --- | --- | --- | --- | --- | --- | --- |
|  | Low | Middle | High | Low | %RSD | Middle | %RSD | High | %RSD |
| Sildenafil | 0.05 | 0.4 | 0.8 | 81 | 8 | 91 | 3 | 92 | 1 |
| Tadalafil | 0.05 | 0.4 | 0.8 | 91 | 3 | 98 | 2 | 100 | 2 |
| Vardenafil | 0.05 | 0.4 | 0.8 | 91 | 7 | 92 | 6 | 93 | 8 |

[Surrogate recovery, n = 3, 0.04 ng mL^-1^] Sildenafil-d_3_ (91.6%±5.2%)


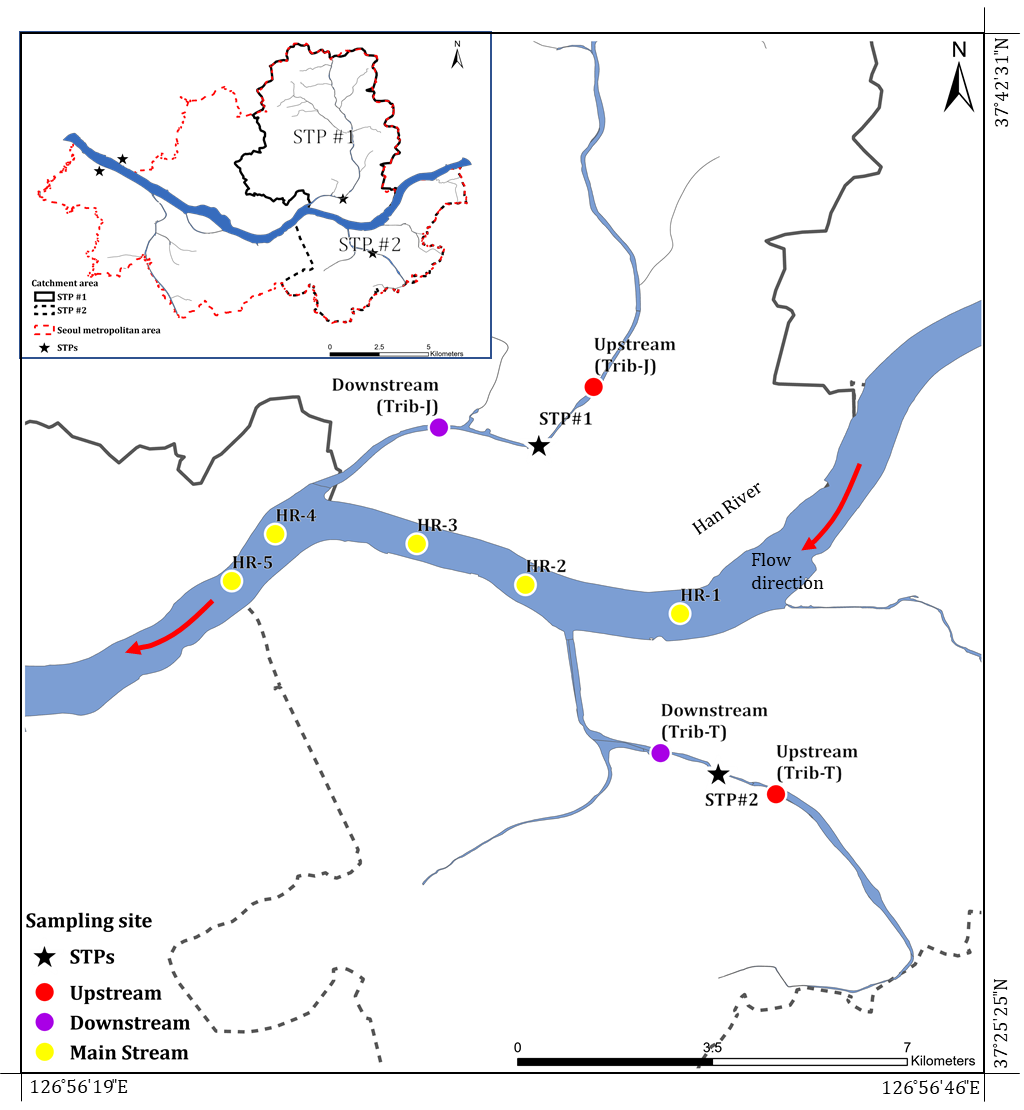


Fig. S1 Sampling sites in this study: influent and effluent of STP#1 and STP#2 (denoted by star dots) and five sites along the Han River (denoted by five yellow circles). Map obtained from the National Geographic Information Institute of Korea (http://map.ngii.go.kr).

**Fig. S2** **Comparison of the daily variation for the mass loadings of PDE-5i considered hydraulic flow rate from STPs.**

Fig. S3 Biodegradability test (batch assay) of Sildenafil and Tadalafil in activated sludge system.

**Fig. S4 Water quality parameters in a) STP#1 and b) STP#2.**

- Pump A; 0.1% acetic acid in water (eluent A for column separation)

- Pump B; acetonitrile (eluent B for column separation)

- Pump C; 0.1% acetic acid in water (eluent C for loading the sample into the SPE)

- Pump D; 0.1% acetic acid/acetonitrile/methanol/isopropanol (1/1/1/1, v/v/v/v, for system cleaning)

**Fig. S5. Configuration of on-line SPE-LC-MS/MS system used in this study.**


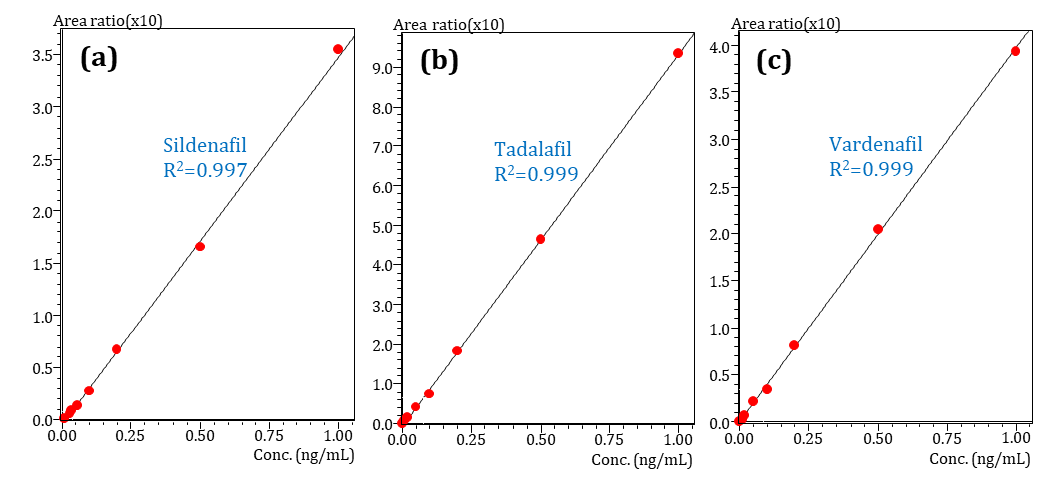


**Fig. S6 Calibration curves of (a) Sildenafil, (b) Tadalafil, and (c) Vardenafil at a range of 0.01-1.0 ng mL^-1^ (calibration point; 0, 0.01, 0.02, 0.05, 0.1, 0.2, 0.5, 1.0 ng mL^-1^).**

**Reference:**

1. Gobry V, Bouchard G, Carrupt P-A, Testa B, Girault HH. Physicochemical Characterization of Sildenafil: Ionization, Lipophilicity Behavior, and Ionic-Partition Diagram Studied by Two-Phase Titration and Electrochemistry. *Helv Chim Acta* **83**, 1465-1474 (2000).
2. Marvin was used for drawing, displaying and characterizing chemical property, Marvin 17.21.0, ChemAxon (https://www.chemaxon.com)
